# A New Texturing Approach of a Polyimide Shielding Cover for Enhanced Light Propagation in Photovoltaic Devices

**DOI:** 10.3390/nano12183249

**Published:** 2022-09-19

**Authors:** Iuliana Stoica, Raluca Marinica Albu, Camelia Hulubei, Dragos George Astanei, Radu Burlica, Gaber A. M. Mersal, Tarek A. Seaf Elnasr, Andreea Irina Barzic, Ashraf Y. Elnaggar

**Affiliations:** 1 Department of Physical Chemistry of Polymers, “Petru Poni” Institute of Macromolecular Chemistry, 700487 Iasi, Romania; 2 Faculty of Electrical Engineering, “Gheorghe Asachi” Technical University of Iasi, 700050 Iasi, Romania; 3 Department of Chemistry, College of Science, Taif University, P.O. Box 11099, Taif 21944, Saudi Arabia; 4 Department of Chemistry, College of Science, Jouf University, Sakaka P.O. Box 2014, Saudi Arabia; 5 Department of Food Science and Nutrition, College of Science, Taif University, P.O. Box 11099, Taif 21944, Saudi Arabia

**Keywords:** polyimide, multi-directional rubbing, morphology, adhesion, illuminance, solar cells

## Abstract

The efficiency of photovoltaics (PVs) is related to cover material properties and light management in upper layers of the device. This article investigates new polyimide (PI) covers for PVs that enable light trapping through their induced surface texture. The latter is attained via a novel strategy that involves multi-directional rubbing followed by plasma exposure. Atomic force microscopy (AFM) is utilized to clarify the outcome of the proposed light-trapping approach. Since a deep clarification of either random or periodic surface morphology is responsible for the desired light capturing in solar cells, the elaborated texturing procedure generates a balance among both discussed aspects. Multidirectional surface abrasion with sand paper on pre-defined directions of the PI films reveals some relevant modifications regarding both surface morphology and the resulted degree of anisotropy. The illuminance experiments are performed to examine if the created surface texture is suitable for proper light propagation through the studied PI covers. The adhesion among the upper layers of the PV, namely the PI and transparent electrode, is evaluated. The correlation between the results of these analyses helps to identify not only adequate polymer shielding materials, but also to understand the chemical structure response to new design routes for light-trapping, which might significantly contribute to an enhanced conversion efficiency of the PV devices.

## 1. Introduction

Solar energy can be regarded as one of the best alternatives to resolve the predicament in the energy sector. The perfecting of the current photovoltaic (PV) technologies is crucial for fulfilling the future energy necessities and also for bypassing the environmental issues, arising from the utilization of the traditional fossil fuels [1]. The introduction of the thin film technology concepts in this domain led to the second generation of PV devices, which are cheaper, since they involve a simple production process and small material consumption [2]. Even if thin-film PVs (TFPVs) are less powerful than silicon-based devices, they are less heat-sensitive and are adequate for use in wide and flat areas, where diffused or weak lighting occurs. As a function of the pursued application, PVs are fabricated in either a “substrate” or a “superstrate” configuration [3]. In the latter version, the solar radiations enter in the PV cell via glass substrate and such design has the advantage of the low-cost encapsulation of solar modules, being suited for high-efficiency tandem devices that are employing the whole solar spectrum for the PV conversion [3].

Some of the current concerns in raising the performance of the TFPVs in “superstrate” configuration reside in overcoming the drawbacks related to the outer cover glass. This component is rigid, thick (~100 microns) and weighs around 70% of the whole PV. Moreover, its low refractivity determines total internal reflection (TIR) losses at the interface with the transparent electrode. This aspect narrows the amount of radiations reaching the active zone and, therefore, the PV cell efficiency is diminished. Based on these aspects, polymers appear to be a better alternative to the classical cover glass, owing to their light weight, flexibility, facile processing and adaptable optical properties via chemical structure (transparency in the desired wavelength interval and refraction matching). Aside from this, thermal stability is mandatory for ensuring polymers cover resistance to long-time exposure to solar radiation heating. Polyimides (PIs) seem to be highly recommendable for this application, since they meet all the above criteria. Some reports describe the PIs utility in PV domain as flexible supports and for this commercial structures are used [4,5,6], while few recent studies discuss the suitability of PIs containing chalcogen atoms and/or alicyclic moieties as covers for solar cells [7,8,9,10]. However, these investigations do not refer to PI surface modification in relation to improvement of the PV cell conversion characteristics via light manipulation through morphology.

To further increase the efficiency of TFPVs close attention must be ascribed to the management of entering solar radiations, since they are responsible for electron-hole pair generation. Generally, there are three methodologies proposed for light trapping in PVs: (a) texturing of the surface morphology, (b) metal nano-inclusions rendering plasmonic scattering, and (c) photonic crystals (Bragg stacks) [11]. These approaches are aiming towards photon trapping and absorption among the upper layers of the PV cell, minimization of the reflection phenomena at the front interfaces and effective utilization of radiations spectrum in a large wavelength domain. One of the most common techniques used for light trapping consists in surface texturing of certain component layers. In this way, diffuse scattering of the incident solar radiations occurs (i.e., the angular distribution of the incoming rays becomes randomized). This determines prolongation of the photons optical paths, in comparison with planar surfaces (lacking texture) and, consequently, an enhanced light capturing is achieved. There are studies that are focused on light management using surfaces with periodic [12] or random structures [13]. However, which type of texture is more efficient for solar radiation trapping is still under debate. A significant difference among the periodic and random structures resides in the fact that the first one is exciting a well-defined Fourier spectrum, coupling radiations solely at particular wavelengths. The second one is displaying a more complex Fourier spectrum, thus enhancing the number of available diffraction orders and hence the amount of photonic states [14,15]. This feature upgrades the photon coupling into a broad spectral domain as a result of radiation scattering in numerous directions [16]. The preplanned optical impact of a randomly textured surface is to reduce optical losses at the entrance facet and also to scatter radiations while increasing photons optical paths. Therefore, the investigation of disordered structures has attracted a special interest, since they are facile to produce, while they diminish the sensitivity to radiation polarization and incidence angle [16,17]. The ordered textures determine sharp peaks in the absorption spectrum generated by coherent resonant Bloch modes, while disordered ones lead to modes superimposing and peak fading, and hence inducing the smoothing of the absorption spectrum (which turns wider and more uniform) [18]. The elucidation of the optimal level of disorder into a structure is complicated. Relatively recent reports found that optimal morphology is neither perfectly ordered nor totally random but a mixture of both categories [19,20]. Among the procedures to fabricate textures, one can mention: hot-embossing method [21], UV nanoimprint lithography [22], nanosphere lithography [23] and other microfabrication routes [24]. Few studies are focused on texturing of the classical glass cover by means of the aforementioned methods for better light management [24,25,26].

Another important aspect of surface texturing resides in the creation of surface roughness, which is known to diminish the light reflectivity [27]. In the case of polymers, surface roughness can be changed by chemical or physical approaches. In the first category, chemical etching is one of the most common techniques that involve the utilization of specific substances to chemically remove certain parts of the material surface [28]. There are several physical methods useful for producing surface roughness, such as plasma exposure, laser irradiation, dynamic plowing lithography and mechanical rubbing. Plasma is known to isotropically corrode and activate the polymer surface within nanometer superficial layers [29]. Although after a certain time the material surface chemical characteristics suffer modifications, the morphology and roughness keep their features. Laser irradiation may induce random or directional (if phase mask is used) surface ablation (material losses) or surface reorganization under ablation threshold [30], but this method is limited to photosensitive polymer structures. Moreover, surface roughness can be attained at nano-scale by dynamic plowing lithography, which involves the creation of nano-traces with atomic force microscope tip in dynamic mode with the custom degree of order/disorder [31]. The technique enables production of pre-defined patterns via raster or vector methods but is constrained to a small surface area. Mechanical abrasion can be performed by scratching the polymer film with materials of distinct toughness, such as textile materials [32] or sandpaper [33], resulting unidirectional micro-grooves and surface anisotropy. In a previous piece of work, we demonstrated that the size and flexibility of the textile fiber is affecting the resulted traces uniformity and dimensions [34]. Literature also shows that the granulation of and the type of the sandpaper is influencing the deepness of the texture [35]. The mechanical approach has the advantage of being a facile, low cost and applicable on larger areas than the aforementioned methods and perhaps this is the reason that is used in many industries, such as liquid crystal displays. Given the benefits introduced by abrasion, multi-directional rubbing is employed to develop a balanced morphology in terms of isotropy/anisotropy, which might be helpful for adequate light trapping. 

This article is a continuation of previous works on solar cell covers based on PIs containing cycloaliphatic units and chalcogen atoms [8,9,36]. Here, we proposed a novel texturing strategy that involves multi-directional rubbing, followed by plasma exposure. The changes in morphology and luminous flux passing through the PI surface are monitored in relation to each step of surface treatment. Moreover, interfacial adhesion of the PI with a transparent electrode is evaluated. These results allow the elucidation of the chemical structure relation with the proposed design routes for light-trapping and might significantly redoundto a larger conversion efficiency of the PV device.

## 2. Materials and Methods

### 2.1. PI Synthesis and Film Preparation

The studied PI structures were attained based on a previously published procedure [9]. Briefly, each polymer was prepared by two-stage polycondensation reaction of bis [4-(4-aminophenoxy) phenyl] sulfone (pBAPS) with either 1,2,3,4-cyclobutanetetracarboxylic dianhydride (CBDA), or 5-(2,5-dioxotetrahydrofuryl)-3-methyl-3-cyclohexene-1,2-dicarboxylic anhydride (EPI) in N-methyl-pyrrolidone (NMP). The film samples were achieved by placing the PI precursor solution on glass supports and then subjected to heating to perform cyclodehydration in a vacuum oven. The films are removed from the glass slides by soaking in water and drying.

### 2.2. Surface Modification by Multi-Directional Rubbing and Plasma Treatment

**Figure 1:**
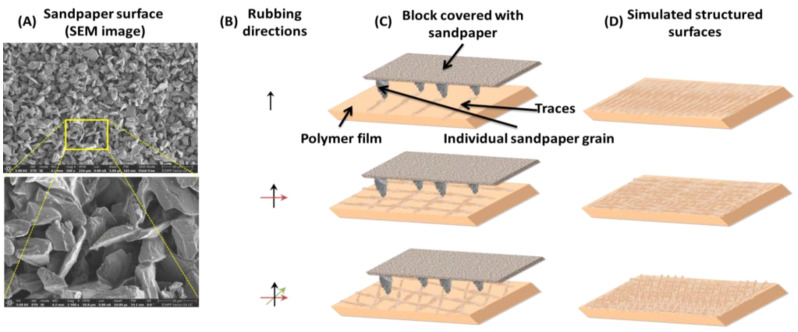
Illustration of the rubbing process: (**A**) SEM images of the sandpaper evidencing the individual grains layout and appearance. The yellow box represents the zone where the sandpaper was scanned at higher magnitude; (**B**) rubbing directions; (**C**) schematic representation of the rubbing device; (**D**) simulated structured polymer surfaces.

Surface treatment was done using a device based on a stand on which the polymeric films are immobilized and rubbed using a parallelepiped covered with 2000 grit sandpaper, which had the velocity of the lateral movement of 5 mm/s. The pressure of the sandpaper on the sample was 2038 Pa, as dictated by 260 g weight of the metallic parallelepiped with the length of 5 cm, width of 2.5 cm and height of 3 cm. In order to investigate the layout and the appearance of the individual sandpaper grains, the surface of the sandpaper was investigated using scanning electron microscopy (SEM) technique, on a Verios G4 UC field emission scanning electron microscope, at an acceleration voltage of 5 kV. The high resolution morphological SEM images presented in Figure 1A, point out that the granular structure of the sandpaper, consisting of silicon carbide, which has a hard, sharp-edged structure. Presented in Figure 1B,C is the schematic representation of the rubbing method made in one direction, in two mutually perpendicular directions, and finally in three directions (two mutually perpendicular directions and the third one at 45 degrees), as indicated by means of black, green and red lines and also the simulated resulted structured surfaces (Figure 1D). The pressure acting on the samples was determined by the parallelepiped weight.

**Figure 2:**
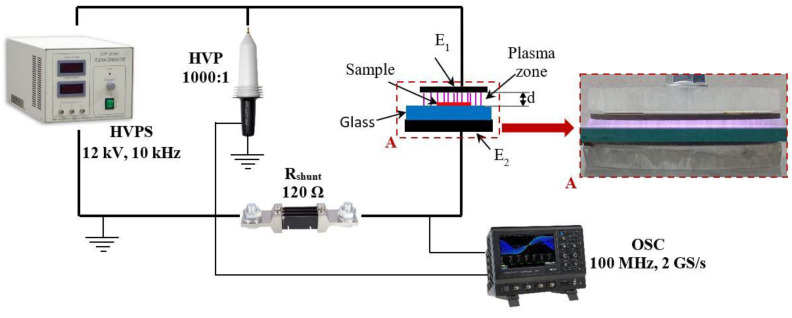
Schematic diagram of experimental set-up.

The three-directional rubbed samples are placed on the surface of the glass sheet, in the middle of the plasma generation zone, and exposed to dielectric barrier discharge (DBD) plasma for two minutes. The plasma was generated in air, at atmospheric pressure and humidity, using a simple reactor configuration, as shown in Figure 2. The reactor was composed by two coplanar electrodes, E_1_ and E_2_, made of stainless steel and having circular shapes with a diameter of 70 mm. The distance between the two electrodes is around 9 mm, including a 6 mm glass sheet as dielectric that covers the ground electrode E_2_, and a 3 mm air gap serving as plasma generation zone.

**Figure 3:**
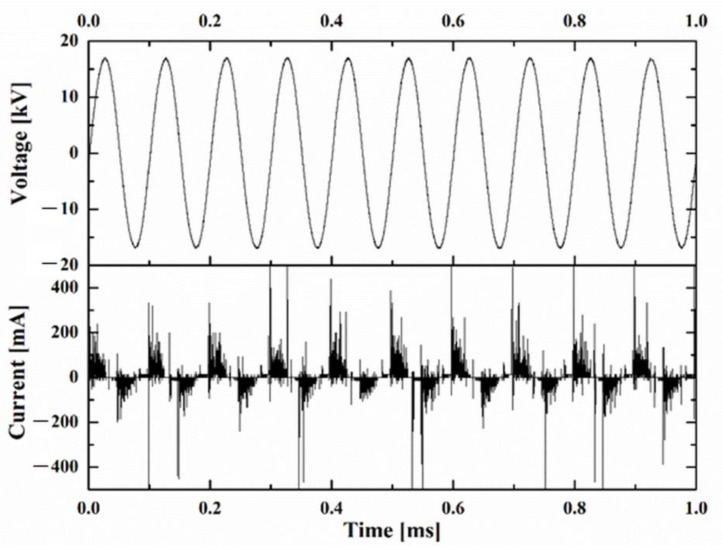
Discharge voltage and current waveforms.

The reactor was supplied using a dedicated plasma generation high voltage power supply (HVPS)—CTP 2000 K. The power supply assured the possibility of adjusting the AC voltage (up to 30 kV) and the frequency (1–100 kHz) delivering a power of up to 500 W. The electrode E_1_ was connected to high voltage output of the power supply, while the E_2_ electrode was connected to the ground. For this experiment, the values of voltage are in the range of 12 kV with the frequency of 10 kHz, while the discharge power was in the range of 40 W. The waveforms corresponding to the voltage and current of the discharge (Figure 3) were recorded using a Lecroy Wavesurfer 3014z. For voltage measurement, a Testec HVP-2739 high voltage probe with a ratio of 1000 to 1 was used—HVP, while the current was measured using a 120 Ω shunt resistance—R_shunt_.

The names and description of the pristine and modified PI samples are given in Table 1.

### 2.3. Characterization

The surface morphology of the pristine samples was investigated by means of atomic force microscopy (AFM) technique using a NTEGRA scanning probe microscope device from NT-MDT Spectrum Instruments (Zelenograd, Moscow, Russia). The acquisition of the height mages was made in tapping mode, with a rectangular shape NSG10 cantilever (length = 95 ± 5 μm; width = 30 ± 3 μm; thickness = 2 ± 0.5 μm, tip height = 14–16 μm; tip curvature radius = 10 nm) with resonant frequency of 352 kHz, in atmospheric conditions at ambient temperature (23 °C). The scanning areas were 10 × 10 μm^2^ and 40 × 40 μm^2^. The texture was characterized using Image Analysis 3.5.0.19892 software by NT-MDT Spectrum Instruments (Zelenograd, Russia) and MountainsLab^®^Premium 9.1 (2022) surface analysis software by DigitalSurf (Besançon, France).

The illuminance data of the initial and surface textured film samples are collected on a CL-70F instrument.

Wettability is determined by means of contact angles experiments performed with a lab-designed instrument. The instrument has a video-based component and a Hamilton syringe. Tests repeated five times involved putting liquid drops of water and ethylene glycol on the PI surface to evaluate the surface tension components.

## 3. Results and Discussion

In order to improve the efficiency of a PV cell, the material used as cover must exhibit adequate surface properties to ensure good adhesion at the interface with electrode and proper light-trapping properties. In the next section of the article, a detailed morphological analysis is performed, together with illuminance and wettability experiments to examine the suitability of the generated surface features onto the PI samples for solar radiation trapping purposes.

### 3.1. Morphology

**Figure 4:**
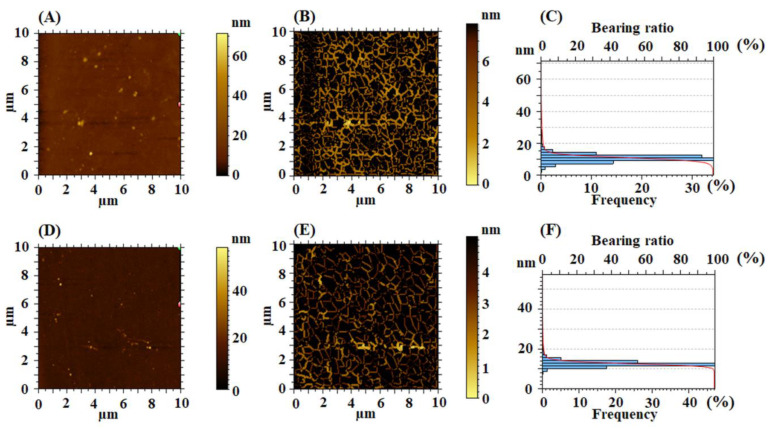
2D topographical images, furrow maps and histograms/Abbott curves obtained for pristine EPI-pBAPS (**A**–**C**) and pristine CBDA-pBAPS (**D**–**F**) samples.

It is known that the polymer morphology affects the optical properties, including the material ability to interact with electromagnetic radiations [37]. For this reason, a deep investigation of the PI morphological evolution with each step of surface modification is made. Since, currently, it yet to be established if the random or periodically textured surface determines improved light trapping in PVs, AFM technique is employed as an advanced tool to extract significant information on the characteristics of the PI surface morphology, and the spatial and functional properties derived from them. Thus, the surfaces of the starting polymers are initially investigated. In Figure 4, the AFM bi-dimensional topographical images of the pristine EPI-pBAPS and CBDA-pBAPS samples are presented, along with the corresponding furrow maps and overlayed height histograms and Abbot–Firestone curves.

The height and hybrid parameters calculated from the AFM measurements are displayed in Table 2. It can be observed that the film surface obtained from the PI derived from the non-symmetrical, bulky and half-flexible alicyclic EPI residues and pBAPS units (which presents in its structure ether links and rigid phenyl rings with a sulfone flexible group between them) has a higher root mean square roughness (Sq) than those obtained for the PI film surface derived from the CBDA (which is a symmetrical, small and rigid moiety) and the same pBAPS segment. This is due to EPI-pBAPS higher degree of freedom, compared to the one of the CBDA-pBAPS sample, induced by the differences in their structures [9]. Using other statistical 3D texture parameter, the entropy of the morphology (Sent), it was possible to quantify the shape-size richness of the ordered or disordered morphology, in correlation with the surface roughness [38]. The values of Sent were around seven, higher for the sample EPI-pBAPS with a higher roughness. This indicates that, for the pristine samples, a relatively smooth morphology, with a lack of richness and diversity. The increased flatness of the CBDA-pBAPS film is also confirmed by the very low value (close to 0%) of the surface area ratio (Sdr). This parameter indicates the complexity degree of the morphology. In our case, the unmodified samples do not show significant deviations from the straight plane. To illustrate the evolution of the surface morphology during the applied modification processes, it is necessary to examine the surface at certain different magnitudes, with extra details being noticed when the scale of examination decreases [39]. In this context, fractal analysis was carried out by using the enclosing boxes method—representing the number of the enclosing boxes as a function of the scale analysis. In this way, the fractal dimension (Df) is calculated. In addition, the scale-sensitive analysis is applied using the graph of the complexity in function of the scale to calculate the fractal complexity (Lsfc). The data regarding these parameters are presented in Table 2. The unmodified EPI-pBAPS sample presents higher fractal dimension correlated with higher fractal complexity than the unmodified CBDA-pBAPS sample. The very low values under 0.40 obtained for the Lsfc (the quantitative measure of the complexity of shape) suggest that the samples have poor surface relief, dominated by low local roughness and smoothness of the edges.

In order to complete the morphological characterization, the furrows analysis is applied. Furrows maps (Figure 4B,E) are generated and from them, parameters, such as the maximum depth of furrow (Fd_max_), mean depth of furrow (Fd_mean_) and mean density of furrows (Fρ_mean_), are calculated and their values are placed in Table 3. These parameters are estimated in topography according to Lemesle et al. [40] as the number of deep lines or scratches detected by patterns in curvature per unit area. The detection and characterization of the furrows from all over the surface indicated that Fd_max_, Fd_mean_ and also Fρ_mean_ are influenced by the surface roughness, being higher for the rough unmodified sample with higher flexibility (EPI-pBAPS). The areal feature parameters, such as mean dale area (Sda) and mean hill area (Sha) [41], are calculated on isolated areas of the surface (i.e., hills and valleys), separated by watershed segmentation method [42,43,44,45] and displayed in Table 3. The relevance of this approach resides in the fact that, based on functional requirements of the surface, it enables the formulation of relationships between peak and valley locations and pursued criteria. It can be observed that the pristine polymers have reduced area covered with hills formations (indicated by the very low values of the Sha), predominantly being the bas-relief (Sda > Sha).

**Figure 5:**
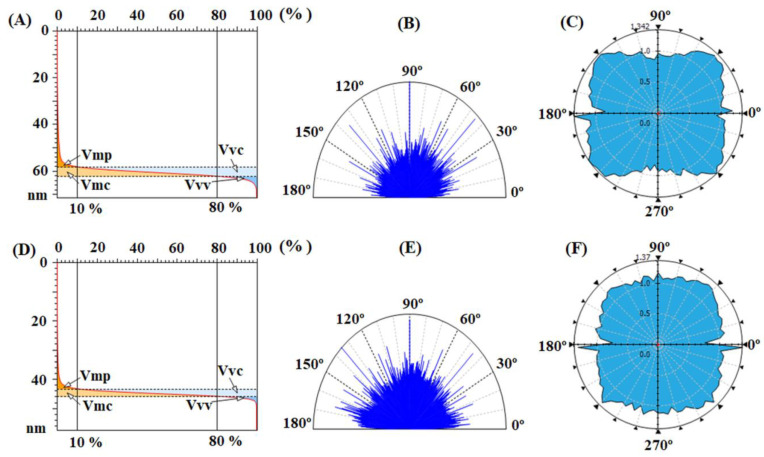
Abbott–Firestone curves with functional volume parameters, texture direction representations and graphs with polar coordinates to analyze orientation with azimuth angle obtained for pristine EPI-pBAPS (**A**–**C**) and pristine CBDA-pBAPS (**D**–**F**) samples.

Related to that, the functional parameters, calculated based on the height histograms from Figure 4C,F and Abbott–Firestone curves from Figure 5A,D and presented in Table 4, are characterized by the functional behavior of the surface, giving acquaintance to the development of the material and the void volumes of which the surface texture is composed. From this point of view, the pristine samples, especially CBDA-pBAPS, have weak-bearing properties, their low morphological characteristics not allowing a proper adhesion to adjacent materials, such as ITO or ZnO layer, for photovoltaic structure.

The balance between the surface morphology isotropy (when the surface is resemblant in every direction) and anisotropy (when the surface has different characteristics in different directions) is determinative for the light-trapping phenomena in solar cells. In order to analyze the texture direction and orientation, the Fourier transform is used to point out the dominant surface directions on polar plots (Figure 5B,E), and the azimuth angle graph in polar coordinates is applied to analyze the orientation of every triangular tile constituent of the investigated surface, by showing the distribution of values for the azimuth angle (beta) (Figure 5C,F). By analyzing both graphs, it is observed that the surface formations do not have a predominant direction of placement on the scanned area, being disposed in all directions. The values of the texture direction index parameter (Stdi from Table 4), which is used to quantify the spatial characteristics, indicate for the pristine polyimides random surface morphologies, CBDA-pBAPS sample being the most isotropic one (having the value closest to 1 for Stdi). These aspects are not compatible with the light trapping on the investigated pristine PI surfaces.

**Figure 6:**
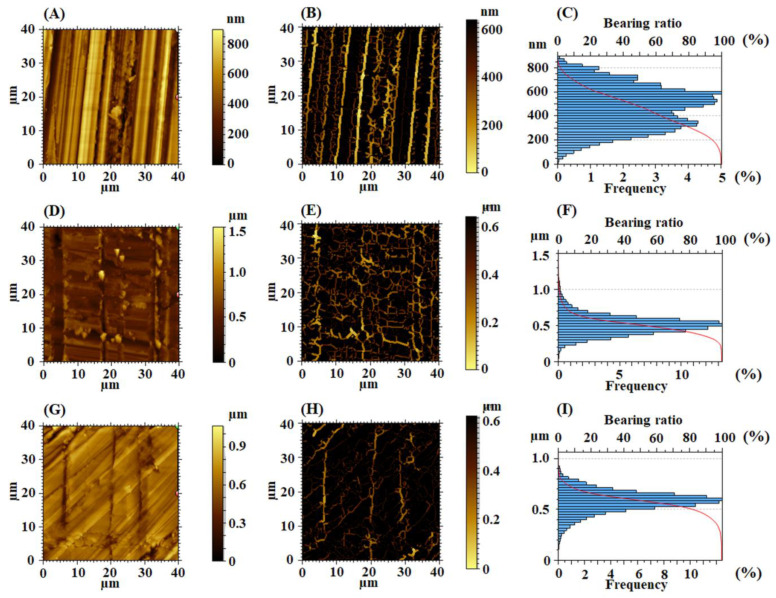
2D topographical images, furrow maps and histograms/Abbott curves obtained for rubbed EPI-pBAPS (1R) (**A**–**C**), EPI-pBAPS (2R) (**D**–**F**) and EPI-pBAPS (3R) (**G**–**I**) samples.

**Figure 7:**
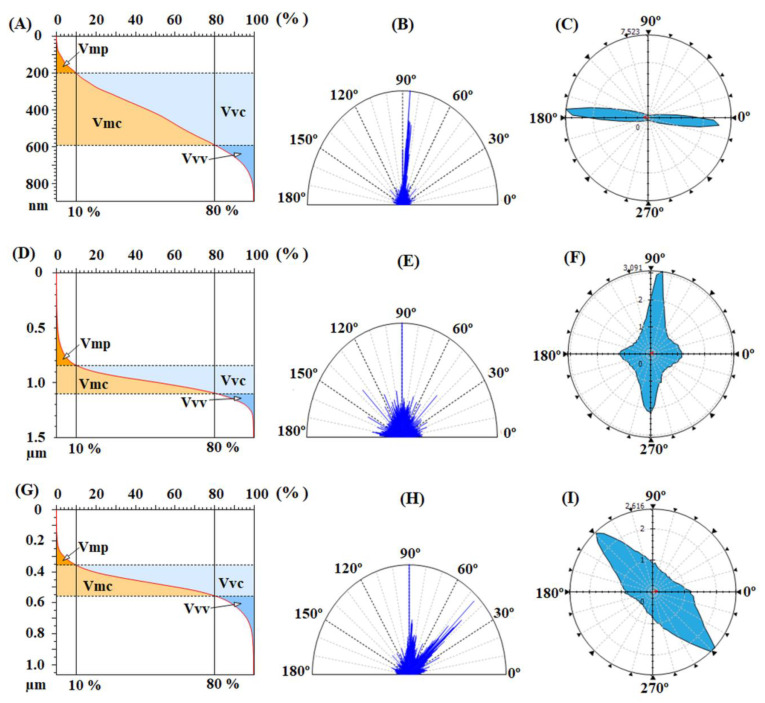
Abbott–Firestone curves with functional volume parameters, texture direction representations and graphs with polar coordinates to analyze orientation with azimuth angle obtained for rubbed EPI-pBAPS (1R) (**A**–**C**), EPI-pBAPS (2R) (**D**–**F**) and EPI-pBAPS (3R) (**G**–**I**) samples.

**Figure 8:**
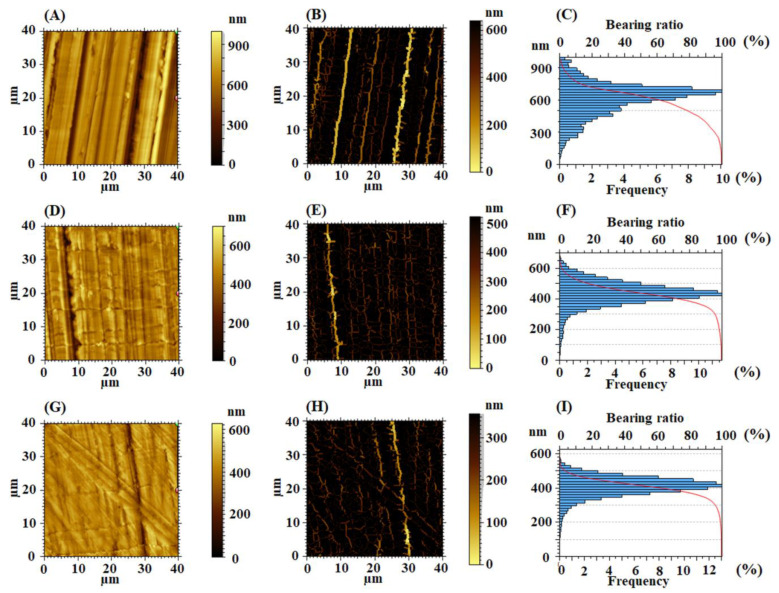
2D topographical images, furrow maps and histograms/Abbott curves obtained for rubbed CBDA-pBAPS (1R) (**A**–**C**), CBDA-pBAPS (2R) (**D**–**F**) and CBDA-pBAPS (3R) (**G**–**I**) samples.

**Figure 9:**
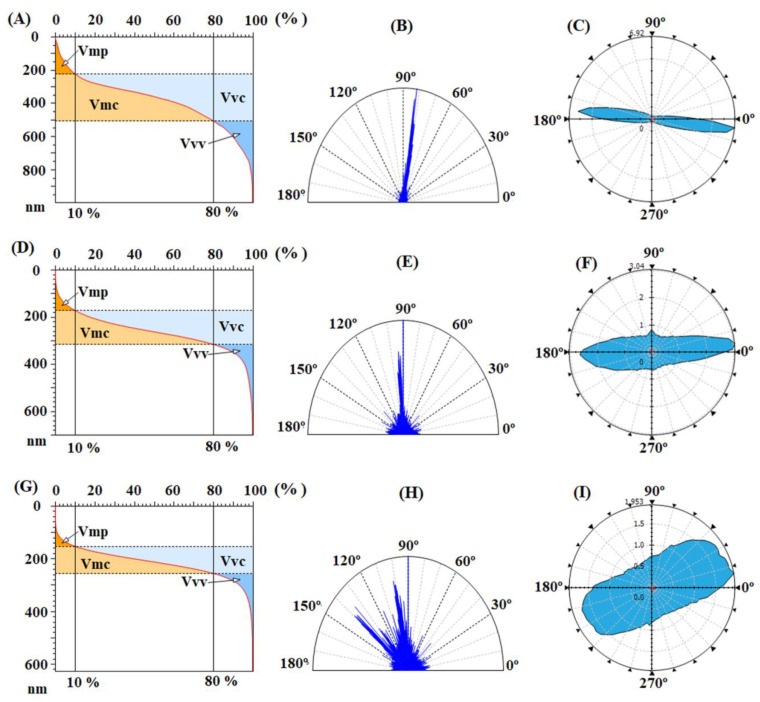
Abbott–Firestone curves with functional volume parameters, texture direction representations and graphs with polar coordinates to analyze orientation with azimuth angle obtained for rubbed CBDA-pBAPS (1R) (**A**–**C**), CBDA-pBAPS (2R) (**D**–**F**) and CBDA-pBAPS (3R) (**G**–**I**) samples.

Since the surface characteristics of the pristine samples themselves do not meet the morphological requirements to be suitable candidates for achieving proper light management in solar cells, the next multidirectional surface abrasion with sand paper on one, two and even three directions is applied. The evolution of the surface texture of the samples, as a consequence, of the rubbing processes and of the properties derived from them is presented in Figure 6 and Figure 7 for the EPI-pBAPS sample and in Figure 8 and Figure 9 for the CBDA-pBAPS sample. Various attributes of the dynamic surface topography can be properly characterized by correctly selecting the parameters describing the structure of the rubbing tools at the macro level and the micro topography of a distinct abrasive grain [39]. From the literature, it is known that a large grit size number indicates a small size of abrasive particles [35]. In this context, the fine sandpaper of 2000 grit size is preferred in order to avoid or to decrease the amount of material removed from surfaces during the unidirectional micro-structuring process. Moreover, silicon carbide is preferred to be used as abrasive synthetic mineral because, compared to the aluminum oxide, it individually has the sharper grain and better shape (see Figure 1A), and several sharp edges can be manipulated concomitantly, in order to clearly pattern the polymer film surface and to provide a better result.

Analyzing the topography resulted after the rubbing processes visually it can be observed that the features from the surface layer of the polymer become oriented in the sand glass friction direction. Although the same pressure and traction rate are maintained during the rubbing process, the behavior of each of the two studied polymers is different, depending on its chemical structure, and thus on the flexibility of the polymer chain derived from it. In this context, the PI material is entangled by the granules on the sandpaper surface and displaced in its direction of movement. In the case of the EPI-pBAPS polymer on the AFM height image (such as in Figure 6A,D,G), well-defined traces and also some uncommon traces with irregularities and defects produced by the material displacement during the rubbing process, causing a real micro-fuzziness, can be observed. This can be explained taking into account the semi-flexible character of the polymer chain.

Roughness is commonly employed as the essential quality measure of the rubbing process, in order to investigate the asperity shapes of the modified surfaces. This phenomenon of material displacement and in some areas its agglomeration into small bumpy formations lead to a considerable increase of the root mean square roughness from ~3 nm obtained for the pristine sample to ~170 nm obtained for the EPI-pBAPS unidirectional rubbed (Table 2). As the rubbing directions increase (2, 3), Sq decreases significantly till ~106 nm for three-directional rubbed sample as a result of the attenuation of the initial traces. On the other hand, on CBDA-pBAPS surface the repetitive marks are homogenous, without any inconstancy (Figure 8A,D,G). Due to its higher rigidity, the roughness induced by the presence of the traces in lower (as seen analyzing the roughness values from Table 2), compared with the EPI-pBAPS sample (~152 nm for the unidirectional rubbing). Instead, the same trend of decreasing the roughness during the additional rubbing direction is present. Regarding the entropy of morphology, Sent parameter (Table 2) doubles during rubbing processes, as compared to the unmodified polymers and slightly decreases for each sample as the number of friction directions increases. For CBDA-pBAPS, the entropy is a little smaller for the same conditions because the traces are more orderly. The values calculated for the surface area ratio (Sdr in Table 2) indicate that the complexity of the morphology is closely influenced by the aspect of the surface features and roughness, following their tendency. Thus, the topography of the rubbed EPI-pBAPS samples is more intricate than that obtained for the rubbed CBDA-pBAPS samples. In addition, as the samples are structured in more directions, the complexity of the morphology decreases significantly. Another tool used to quantitatively measure the complexity in modulated relief development is the fractal analysis (Table 2). As the abrasion process develops in one direction, the traces became larger and more complex in shape, this aspect being translated in a rapidly increasing value of the Lsfc. On the other hand, as the directions of rubbing multiply, the fractal complexity decreases, while the fractal dimension increases. This can be considered a sign of the 3D development of the morphology. EPI-pBAPS sample presents higher fractal complexity at each stage of rubbing, compared with CBDA-pBAPS polymer, suggesting that these structured surfaces can be characterized by a richer relief, due to its higher flexibility.

The real aspect of the traces from the region of the relief valleys is highlighted through the bright colors from the furrow maps (Figure 6B,E,H and Figure 8B,E,H), and the corresponding parameters are calculated during furrows analysis (Table 3). Compared with the pristine samples, the density of furrows decreases 4–5 times, as may be the case. As expected, the maximum and mean depth of furrow shows higher values for the rubbed EPI-pBAPS sample promoted by the appearance of deeper channels, for each kind of uni-directional, bi-directional and three-directional rubbing. Furthermore, the bi-directional rubbed samples exhibited the highest mean density of furrows because there is a greater distribution of furrows in these samples. Moreover, in this case the superior Fρ_mean_ value of 6124 cm/cm^2^ calculated for CBDA-pBAPS, compared to 5737 cm/cm^2^ for EPI-pBAPS, is due to clearly defined traces discovered on the surface.

Regarding the feature parameters implied in functional requirements of the surface and displayed in Table 3, in the case of the unidirectional rubbing, the mean dale area is determinate by the traces left on the surface of the polymer by the rubbing process. In this context, due to the considerable distance between the traces, Sha parameter will be higher than Sda. Rubbing in two perpendicular directions increases the number of traces, inducing implicitly the increasing of the valleys areas, compared to those corresponding to the hills (Sda > Sha). When the sand paper rubbing process is performed in all three directions, in each case a balance between the high and low regions is observed, and both the dales and the hills occupy similar areas on the investigated surface (Sda ≈ Sha).

Analyzing the height distributions from Figure 6C,F,I for the rubbed EPI-pBAPS and Figure 8C,F,I for the rubbed CBDA-pBAPS, it can be observed that the ampleness of the histograms collected from the uni-directional rubbed samples is higher, as compared to the other rubbing techniques. This is also in agreement with entropy values. The Abbott curves (Figure 7 and Figure 9A,D,G) suggest a bigger difference between the material volumes and the void volumes (Table 4) when one direction of structuring was used, this gap being minimized by additional steps of sanding. The isotropy/anisotropy balance, decisive for the light propagation in photovoltaic devices, was evaluated during the rubbing processes through the texture direction representations (Figure 7 and Figure 9B,E,H) and azimuth angle graph in polar coordinates (Figure 7 and Figure 9C,F,I). The graphs confirm that the surface morphology properties are influenced by the rubbing direction. When the surfaces are unidirectionally modified, the graphs show the orientation of the morphological features in only one direction, and thus higher anisotropy (indicated by the close to zero values of the Stdi parameter from Table 4). This anisotropy is moderately diminished as additional direction of abrasion is added; Stdi grows to higher values but is still under 0.450. The texture developed in polar coordinates is showing the orientation with azimuth angle in two and three directions, respectively. In each direction, the properties are different, preserving the anisotropy of the morphology.

**Figure 10:**
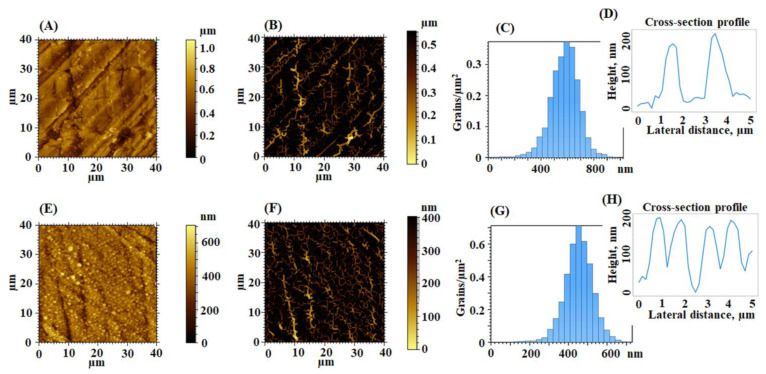
2D topographical images, furrow maps, grains distribution and grains cross-section profiles obtained for plasma-treated rubbed EPI-pBAPS (3R) (**A**–**D**) and CBDA-pBAPS (3R) (**E**–**H**) samples.

Furthermore, the samples that were three-directional rubbed are placed into the plasma generation zone. When the high voltage is applied and it exceeds the breakdown voltage, an ionization phenomenon of the gas between the electrodes occurred, determining the ignition of plasma streamers between the electrodes and the dielectric. The dielectric has the purpose to avoid the streamers to pass into a single channel of arc plasma, maintaining the non-thermal character of the discharge and assuring a homogeneous distribution of the plasma in the plasma zone. In this case, the surface of the sample is bombarded by free electrons and by the reactive species generated into the plasma. The electrical discharge extinguishes when the voltage values became lower than the minimum threshold voltage and reignites after changing the polarity. Analyzing the texture aspect of the surfaces by the instrumentality of the 2D topographical images from Figure 10, it can be concluded that the main morphology obtained when the samples are three-directional rubbed is preserved, but it is enriched with globular nano-sized formations induced by the previously presented phenomena, occurring during plasma irradiation process. Thus, the height characteristics are enhanced, placing the roughness at a higher level. Comparing to the starting three-directional rubbed films, the plasma-treated surfaces have superior Sq and increased richness of the morphology, transposed by the values of the entropy of morphology and surface area ratio presented in Table 2. The grains distribution plots (Figure 10C,G) indicate that the density of the grains is lower for the plasma-treated EPI-pBAPS (3R) and much higher for the plasma treated CBDA-pBAPS (3R). In this case, the granular formations are uniform distributed all over the surface, having average heights of about 140 nm, compared to those seen on EPI-pBAPS, which are higher (of approximately 190 nm) and more randomly spread, mostly on the edges of the three-directional structured relief. These average height values of the granules, calculated from the cross-section profile from Figure 10D,H, are quite small (up to 200 nm), with respect to the wavelength of light (400–700 nm). In this context, mainly the well-dispersed grain-like nanopatterns discovered on CBDA-pBAPS can create a refractive index gradient between air and the adjuvant layer in the PV structure, favoring the reduction in the light reflection [46].

In terms of the fractal complexity of the shape, both parameters derived from the fractal analysis (Table 2) of the small particles. The effect is prevailing once again for the CBDA-BAPS sample. The value of 2.8 for Df (close to 3) achieved for this polymer predicted the existence of three-dimensional fractal geometry on the surface [47] due to the intense action of the dielectric barrier discharge plasma.

**Figure 11:**
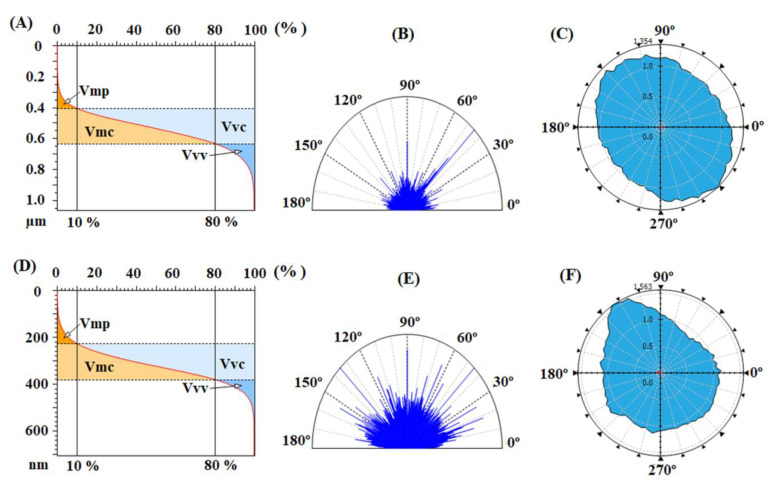
Abbott–Firestone curves with functional volume parameters, texture direction representations and graphs with polar coordinates to analyze orientation with azimuth angle obtained for DBD plasma-treated rubbed EPI-pBAPS (3R) (**A**–**C**) and CBDA-pBAPS (3R) (**D**–**F**) samples.

The contribution of the bean-like structures developed on the plasma treated surfaces is also found in the furrow maps from Figure 10B,F, where the maximum and mean depth of furrow are significantly increased, compared to the values obtained for the samples rubbed in all three directions (Table 3). The highest mean density of furrows was found for CBDA-BAPS sample. Related to that, regarding the feature parameters, during DBD plasma treatment, due to the appearance of the globular formations, the deep valleys and high hills are attenuated, prevailing the dale in the surface relief (Sda > Sha) (Table 3). Compared to the starting three-directional rubbed samples, from the bearing curves (Figure 11A,D), it can be observed that, after plasma, the material volume from the peak zone decreases for the EPI-BAPS due to the arrangement of the grains, especially in/on the boundary of the traces created during rubbing, while increases for CBDA-BAPS are as a consequence of development of the grain-like structures all over the surface. These findings can further explain the increases of the material and void volume in the core zone and the decreases of the void volume in the valley region for both samples (see the data from Table 4). This balancing of the surface from a morphological point of view leads to the decrease in the anisotropy and the development of an incipient isotropy. The mentioned modification can be visually observed in the angular spectra (Figure 11B,E) and the graphs with polar coordinates analyzed orientation with the azimuth angle (Figure 11C,F) and quantitatively evaluated through the values for the Stdi parameter, slightly higher than 0.500 (Table 4). This, correlated with the randomly modulated aspect of the surface, creates, from the point of view of the texture aspect, a favorable framework for improving the light-capturing capacity of the polymer layer.

### 3.2. Wettability and Adhesion

In a PV structure, the PI layer is interfaced the transparent conductive electrode. Materials, such as ZnO or ITO, are often used for the fabrication of electrodes employed in solar cells [48,49]. In order to attain good reliability of the device, it is highly desirable to obtain high adhesion at the cover/electrode interface. In the case of polymers, adhesion interactions can be enhanced by plasma irradiation, followed by electrode deposition. In the case of CBDA-pBAPS and EPI-pBAPS films, one side of the samples is textured, as described above, and the other is exposed for 2 min in plasma to enhance polarity and improve interfacial adhesion with the electrode material. These interactions can be determined based on the surface tension data of the two phases coming into contact, as revealed by Equation (1):

(1) $$W_{\mathrm{ad}}=2\cdot \left(\sqrt{\sigma_{s}^{d}\cdot \sigma_{e}^{d}}+\sqrt{\sigma_{s}^{p}\cdot \sigma_{e}^{p}}\right)$$

where W_ad_—work of adhesion between the PI sample and electrode layer, σ—surface tension, the superscripts “d” and “p” refer to the disperse and polar components, while the subscripts “s” and “e” refer to the sample and electrode, respectively.

To calculate the surface tension components required for W_ad_ estimation, the contact angle values measured on PI films are introduced in the Young relation [50] shown in Equation (2):

(2) $$\frac{\left(1+\cos\theta \right)\sigma_{l}}{2}=\sqrt{\sigma_{s}^{d}\sigma_{l}^{d}}+\sqrt{\sigma_{s}^{p}\sigma_{l}^{p}}$$

where θ—contact angle of water (W) or ethylene glycol (EG) on the PI surface; the subscript “l” refers to the test liquid.

Table 5 lists the experimental data on contact angles on the CBDA-pBAPS and EPI-pBAPS films, before and after plasma irradiation.

The pristine CBDA-pBAPS has slightly higher surface polarity, owing to the presence of small and polar CBDA moieties, which enhance the density of polar imide rings along the main chain. The sample pristine EPI-pBAPS displays prevalent surface dispersive character due to its bulky and semi-flexible cycloaliphatic EPI units. Upon plasma exposure, both PIs gain an increased polar surface tension component, especially CBDA-pBAPS sample. It can be noted that these aspects are influencing the adhesion interactions with the electrode material. The higher polarity of CBDA-pBAPS pristine film favors higher adhesion with the selected conductive oxides than in the case of EPI-based sample. Regardless of the polymer structure, a stronger adhesion is remarked for ITO than for ZnO. Moreover, plasma treatment of the PIs generates an increase in the magnitude of W_ad_ at the interface with the transparent electrodes, especially for CBDA-pBAPS. Based on this, it seems that this PI structure (after plasma exposure) leads to a more reliable PV structure when it is interfaced with an ITO-made electrode.

### 3.3. Illuminance

**Figure 12:**
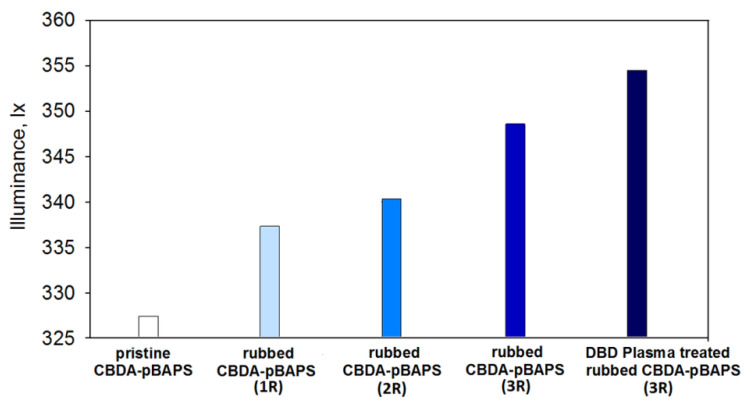
Illuminance variation with each step of the PI surface modification.

The morphological and wettability results indicated that CBDA-pBAPS sample displays better properties than EPI-pBAPS. So, in the next stage of the study, illuminance tests are done only for this PI structure. Figure 12 illustrates the variation of this optical parameter with each step of surface modification of the CBDA-derived sample. The total luminous flux falling through the initial PI film is 329 lx. After creation of the first set of traces (under the form of grooves), the luminous flux is observed to increase to about 337 lx. Additional rubbing of the PI film along two distinct directions determines further enhancement of the luminous flux up to 340 lx and 348 lx, respectively. In the final step, plasma exposure is etching the resulted texture of the three times rubbed polymer foil and this induces a luminous flux increase up to 354 lx.

The propagation of the radiations through rough and smooth interfaces is highly dissimilar due to the scattering phenomenon. For PV applications, the goal is to attain the smallest reflectance to ensure the desired radiation light trapping inside the device layers. When roughness appears at the interface of two media from the PV cell, the radiations are scattered into distinct directions and these beams reach the interfaces under variable angles. This determines larger angular-sensitive reflectance at the interfaces, in comparison to the situation of normal incidence. As a consequence, the trapping of radiations is more efficient, since their path and absorption in the materials from the device supplementary increases [51,52]. On the other hand, the generation of surface roughness not only diminishes the reflectivity but additionally meliorates the wear resistance [27]. Hence, upon the mechanical abrasion of the CBDA-pBAPS, the possibilities of incident radiation to bounce back onto the material surface are increased rather than coming out to the initial medium (air), while the radiation propagation distance is enhanced inside the polymer medium. It is reported that to accomplish better radiation trapping and absorbance in PV device, aside from surface roughness, the surface topography is also essential. In other words, proper light management is based both on scattering at rough interfaces and also on the randomization of the propagation paths by means of the material surface morphological features. So, it is believed that adequate light management is based on a combined effect of surface roughening and texture of surface topography, including its level of disorder. Since the current light management concepts, based on modulated surface textures, involve the idea of modulated texture (mixture of random and periodic) [11], additional rubbing along distinct directions is performed to adapt the sample surface roughness and topography to the pursued scope. Upon each step of rubbing, the surface roughness of the PI film is considerably enhanced in regard to the pristine sample (as shown by Sq parameter). Moreover, the initial isotropic PI surface is changed into an anisotropic one, but the supplementary rubbing is slightly diminishing this aspect (as revealed by Stdi values). In addition, the sub-wavelength features of the generated texture on the rubbed PI surface favor the anti-reflecting effects, as supported by literature [11]. Plasma exposure leads to the occurrence of globular formations of nanometric dimensions on the three-time rubbed CBDA-pBAPS, which increase surface roughness and are randomly distributed on the sample surface (as denoted by Sq and Stdi parameters). Such morphological characteristics might be efficient for proper light coupling, either via Mie resonance to intensify forward radiation scattering [53]. This type of resonance is known as morphology-influenced resonance and is proved to concentrate incident radiations in the nanoparticles [54], and the scattering effect is more pronounced for high refractive index materials [55]. In another work [9], it is shown that the studied polymers display high refractive index. It is known that for small particles, the electric dipole contribution has a higher significance in the scattering cross-section [56]. Thus, it is presumed that when the incident wave falls on the globular formations from the PI surface, having dimensions little above 100 nm, the contribution of the electric dipole to scattering prevails in the optical domain. So, the amount of radiation propagating through the rubbed and plasma-modified sample surface is higher. This surface texturing approach produces the appropriate roughness and topography for proper light scattering and trapping inside the PI cover, as demanded for PV applications.

## 4. Conclusions

This work aimed to test the ability of the proper structuring of two PIs with potential use as covers for solar cells. For this purpose, a new approach of surface adaptation is developed to examine the resulted texture suitability for light trapping in the studied samples used as upper layers in PVs. The texturing method involves multi-directional mechanical abrasion followed by plasma etching. Moreover, to improve the compatibility with the contacting electrode, the opposite side of the samples is plasma irradiated.

AFM investigations highlighted that the PI chain flexibility affects the size and uniformity of the created surface features after each rubbing step. The surface bearing properties are more pronounced when the anisotropy level is highest. Supplementary rubbing determines the gradual increase in surface isotropy. The enhancement of Sq in regard to pristine samples is compatible with reflection minimization at PI/air interface. Plasma exposure of three-time rubbed sample, determine a distinct morphology on each studied structure, namely nano-sized globular formations appear onto the CBDA-pBAPS, while on EPI-pBAPS, they are not visible.

The adhesion of the imide-type polymers with electrodes is larger for ITO, in comparison with ZnO, particularly for CBDA-pBAPS. This is further enhanced upon plasma exposure of the samples, assuring better reliability of the PV device.

The illuminance data indicate that the resulted modulated surface textures lead to increase in luminous flux, showing that both surface roughness and topography favor better light trapping inside the modified PI films in regard to the pristine one. This approach determines proper light scattering and trapping inside the polymer cover for PV cell by minimization of the reflection and absorption losses via combined effects of surface roughening and occurrence of sub-wavelength textures that are distributed with a certain degree of disorder on the sample surface.

## Figures and Tables

**Table 1:** The names and descriptions of the polyimide samples.

Sample Name	Description
EPI-pBAPS	Pristine EPI-pBAPS sample with no surface modification
Rubbed EPI-pBAPS (1R)	Rubbed EPI-pBAPS sample along one direction
Rubbed EPI-pBAPS (2R)	Rubbed EPI-pBAPS sample along two perpendicular directions
Rubbed EPI-pBAPS (3R)	Rubbed EPI-pBAPS sample along perpendicular directions followed by additional rubbing at 45°
DBD Plasma treated Rubbed EPI-pBAPS (3R)	Rubbed EPI-pBAPS sample along perpendicular directions followed by additional rubbing at 45° and then exposed to dielectric barrier discharge plasma
CBDA-BAPS	Pristine CBDA-BAPS sample with no surface modification
Rubbed CBDA-pBAPS (1R)	Rubbed CBDA-BAPS sample along one direction
Rubbed CBDA-pBAPS (2R)	Rubbed CBDA-BAPS sample along two perpendicular directions
Rubbed CBDA-pBAPS (3R)	Rubbed CBDA-BAPS sample along perpendicular directions followed by additional rubbing at 45°
DBD Plasma treated Rubbed CBDA-pBAPS (3R)	Rubbed CBDA-BAPS sample along perpendicular directions followed by additional rubbing at 45° and then exposed to dielectric barrier discharge plasma

**Table 2:** Height, hybrid and fractal parameters calculated from the AFM measurements of the pristine, rubbed along different directions and plasma-treated samples, based on the height histogram and furrow analysis.

Sample	Height and Hybrid Parameters			Fractal Analysis	
	Sq (nm)	Sent	Sdr (%)	Df	Lsfc
Pristine EPI-pBAPS	3.2	7.99	0.23	2.42	0.38
Rubbed EPI-pBAPS (1R)	170.6	13.99	8.77	2.48	19.29
Rubbed EPI-pBAPS (2R)	134.7	13.60	7.26	2.49	13.11
Rubbed EPI-pBAPS (3R)	106.5	13.35	3.82	2.51	7.61
DBD Plasma treated Rubbed EPI-pBAPS (3R)	116.3	13.51	8.14	2.72	14.11
Pristine CBDA-BAPS	1.8	7.28	0.02	2.34	0.23
Rubbed CBDA-pBAPS (1R)	152.1	13.74	4.28	2.42	11.47
Rubbed CBDA-pBAPS (2R)	77.2	12.96	2.64	2.69	6.44
Rubbed CBDA-pBAPS (3R)	56.5	12.51	1.59	2.83	3.33
DBD Plasma treated Rubbed CBDA-pBAPS (3R)	74.9	12.91	6.16	2.85	15.59

Sq: root mean square roughness, Sent: entropy of morphology, Sdr: surface area ratio, Df: fractal dimension, Lsfc: fractal complexity.

**Table 3:** Furrow and feature parameters calculated from the AFM measurements of the pristine, rubbed along different directions and plasma-treated samples.

Sample	Furrow Analysis			Feature Parameters	
	Fd_max_ (nm)	Fd_mean_ (nm)	Fρ_mean_ (cm/cm^2^)	Sda (µm^2^)	Sha (µm^2^)
Pristine EPI-pBAPS	7.2	3.2	25,878	2.2	0.4
Rubbed EPI-pBAPS (1R)	610.9	234.7	5651	6.4	7.1
Rubbed EPI-pBAPS (2R)	603.9	231.0	5736	14.5	9.6
Rubbed EPI-pBAPS (3R)	497.2	159.5	5287	12.9	12.4
DBD Plasma treated Rubbed EPI-pBAPS (3R)	534.1	171.7	5820	5.5	3.1
Pristine CBDA-pBAPS	4.4	2.1	21,520	1.3	0.2
Rubbed CBDA-pBAPS (1R)	609.7	180.0	5311	10.7	20.6
Rubbed CBDA-pBAPS (2R)	472.5	136.9	6124	6.1	5.8
Rubbed CBDA-pBAPS (3R)	352.4	87.2	5802	8.1	8.2
DBD Plasma treated Rubbed CBDA-pBAPS (3R)	368.9	130.9	6407	2.3	1.9

Fd_max_: maximum depth of furrow, Fd_mean_: mean depth of furrow, Fρ_mean_: mean density of furrows, Sda: mean dale area, Sha: mean hill area.

**Table 4:** Functional volume and spatial parameters calculated from the AFM measurements of the pristine, rubbed along different directions and plasma-treated samples, based on the Abbott–Firestone curves and texture direction representations and graphs with polar coordinates, respectively.

Sample	Functional Volume Parameters				Spatial Parameter
	Vmp (µm^3^/µm^2^)	Vmc (µm^3^/µm^2^)	Vvc (µm^3^/µm^2^)	Vvv (µm^3^/µm^2^)	Stdi
Pristine EPI-pBAPS	0.000325	0.00168	0.00237	0.000236	0.644
Rubbed EPI-pBAPS (1R)	0.0057	0.1724	0.2186	0.0154	0.165
Rubbed EPI-pBAPS (2R)	0.0112	0.106	0.148	0.0138	0.419
Rubbed EPI-pBAPS (3R)	0.00544	0.0839	0.113	0.0164	0.424
DBD Plasma treated Rubbed EPI-pBAPS (3R)	0.00521	0.101	0.127	0.0159	0.526
Pristine CBDA-pBAPS	0.000182	0.00103	0.00155	0.000125	0.753
Rubbed CBDA-pBAPS (1R)	0.00772	0.134	0.148	0.0234	0.175
Rubbed CBDA-pBAPS (2R)	0.00364	0.0590	0.0850	0.0113	0.225
Rubbed CBDA-pBAPS (3R)	0.00228	0.0452	0.0577	0.00874	0.315
DBD Plasma treated Rubbed CBDA-pBAPS (3R)	0.00441	0.0636	0.0899	0.00840	0.636

Vmp: Peak material volume, Vmc: Core material volume, Vvc: Core void volume, Vvv: Pit void volume, Stdi: texture direction index of the surface.

**Table 5:** The results regarding the contact angles, dispersive and polar surface tensions of the PIs and their work of adhesion with ITO or ZnO.

Sample	Contact Angle (°)		$\sigma_{s}^{d}$ (mN/m)	$\sigma_{s}^{p}$ (mN/m)	W_ad_ (ITO) (mN/m)	W_ad_ (ZnO) (mN/m)
	W	EG				
CBDA-pBAPS	66	48	12.06	24.00	81.02	43.43
CBDA-pBAPS plasma	31	10	6.32	61.18	99.48	44.90
EPI-pBAPS	73	55	12.75	18.09	75.82	42.18
EPI-pBAPS plasma	60	33	18.16	23.62	88.43	49.66

## Data Availability

Not applicable.

## References

[1] Dambhare M.V., Butey B., Moharil S.V. (2021). Solar photovoltaic technology: A review of different types of solar cells and its future trends. J. Phys. Conf. Ser..

[2] Lee T.D., Ebong A.U. (2017). A review of thin film solar cell technologies and challenges. Renew. Sustain. Energy Rev..

[3] Avrutin V., Izyumskaya N., Morkoç H. (2011). Semiconductor solar cells: Recent progress in terrestrial applications. Superlattices Microstruct..

[4] Yusoff A.R.M.M., Syahrul M.N., Henkel K. (2007). Film adhesion in amorphous silicon solar cells. Bull. Mater. Sci..

[5] Patel D., Deshmukh S.P. (2012). Polymer in Sustainable Energy. J. Miner. Mater. Charact. Eng..

[6] Dayneko S., Tameev A., Tedoradze M., Martynov I., Artemyev M., Nabiev I., Chistyakov A. (2013). Hybrid heterostructures based on aromatic polyimide and semiconductor CdSe quantum dots for photovoltaic applications. Appl. Phys. Lett..

[7] Hulubei C., Albu R.M., Lisa G., Nicolescu A., Hamciuc E., Hamciuc C., Barzic A.I. (2019). Antagonistic effects in structural design of sulfur-based polyimides as shielding layers for solar cells. Sol. Energy Mater. Sol. Cells.

[8] Barzic A.I., Albu R.M., Hulubei C. (2021). Polyimides Containing Chalcogen Atoms in Solution Phase: Viscoelasticity and Interferometry Analyses. Rev. Roum. Chim..

[9] Barzic A.I., Albu R.M., Stoica I., Varganici C.D., Hulubei C. (2022). Polyimides containing cycloaliphatic units and chalcogen atoms as alternative shielding coatings for solar cells. Polym. Bull..

[10] Barzic A.I., Albu R.M., Stoica I., Hulubei C. (2022). New shielding covers based on transparent polyimide/ferrous sulfide composites that reduce optical losses in solar cells. Compos. Sci. Technol..

[11] Zeman M., Isabella O., Jäger K., Santbergen R., Solntsev S., Topic M., Krc J. (2012). Advanced Light Management Approaches for Thin-Film Silicon Solar Cells. Energy Procedia.

[12] Meier M., Paetzold U.W., Ghosh M., van Erven R. (2015). Periodic nano-textures enhance efficiency in multi-junction silicon thin-film solar cells. Phys. Status Solidi.

[13] Kowalczewski P., Bozzola A., Liscidini M., Andreani L.C., Wehrspohn R.B., Gombert A. (2014). Tailoring randomly rough textures for light trapping in thin-film solar cells. Photonics for Solar Energy Systems V.

[14] Martins E.R., Li J., Liu Y., Zhou J., Krauss T.F. (2012). Engineering gratings for light trapping in photovoltaics: The supercell concept. Phys. Rev. B.

[15] Bozzola A., Liscidini M., Andreani L.C. (2014). Broadband light trapping with disordered photonic structures in thin-film silicon solar cells. Prog. Photovolt. Res. Appl..

[16] Muller J., Herman A., Mayer A., Deparis O. (2015). A fair comparison between ultrathin crystalline-silicon solar cells with either periodic or correlated disorder inverted pyramid textures. Opt. Express.

[17] Oskooi A., Favuzzi P.A., Tanaka Y., Shigeta H., Kawakami Y., Noda S. (2012). Partially disordered photonic-crystal thin films for enhanced and robust photovoltaics. Appl. Phys. Lett..

[18] Peretti R., Gomard G., Lalouat L., Seassal C., Drouard E. (2013). Absorption control in pseudodisordered photonic-crystal thin films. Phys. Rev. A.

[19] Lin C., Huang N., Povinelli M.L. (2012). Effect of aperiodicity on the broadband reflection of silicon nanorod structures for photovoltaics. Opt. Express.

[20] Vynck K., Burresi M., Riboli F., Wiersma D.S. (2012). Photon management in two-dimensional disordered media. Nat. Mater..

[21] Haug F.-J., Söderström T., Python M., Terrazzoni-Daudrix V., Niquille X., Ballif C. (2009). Development of micromorph tandem solar cells on flexible low-cost plastic substrates. Sol. Energy Mater. Sol. Cells.

[22] Escarré J., Battaglia C., Söderström K., Pahud C., Biron R., Cubero O., Haug F.-J., Ballif C. (2012). UV imprinting for thin film solar cell application. J. Opt..

[23] Deckman H.W., Dunsmuir J.H. (1982). Natural lithography. Appl. Phys. Lett..

[24] Chen L., Fang B., Ke Q., Wangyang P., Hu K., Zhang W. (2022). Quasi-hemispherical pit array textured surface for increasing the efficiency of thin-film solar cells. AIP Adv..

[25] Park H., Shin M.H., Iftiquar S.M., Hussain S.Q., Ju M., Kim Y., Cho E.-C., Yi J. (2020). The light-trapping effect in various textured cover glass for enhancing the current density in silicon heterojunction solar cells. Opt. Commun..

[26] Park H., Jeong Y., Myunghun L., Lee S.Y., Lee J., Park C. (2018). Effects of Glass Texturing Structure on the Module Efficiency of Heterojunction Silicon Solar Cells. Curr. Photovolt. Res..

[27] Liu H., Du Y., Yin X., Bai M., Liu W. (2022). Micro/Nanostructures for Light Trapping in Monocrystalline Silicon Solar Cells. J. Nanomater..

[28] Kim S.R. (2000). Surface Modification of Poly(tetrafluoroethylene) Film by Chemical Etching, Plasma, and Ion Beam Treatments. J. Appl. Polym. Sci..

[29] Popovici D., Barzic A.I., Stoica I., Butnaru M., Ioanid G.E., Vlad S., Hulubei C., Bruma M. (2012). Plasma modification of surface wettability and morphology for optimization of the interactions involved in blood constituents spreading on some novel copolyimide films. Plasma Chem. Plasma Process..

[30] Stoica I., Epure E.L., Constantin C.P., Damaceanu M.D., Ursu E.L., Mihaila I., Sava I. (2021). Evaluation of local mechanical and chemical properties via afm as a tool for understanding the formation mechanism of pulsed uv laser-nanoinduced patterns on azo-naphthalene-based polyimide films. Nanomaterials.

[31] Stoica I., Barzic A.I., Hulubei C. (2017). Fabrication of nanochannels on polyimide films using dynamic plowing lithography. Appl. Surf. Sci..

[32] Yamahara M., Nakamura M., Koide N., Sasaki T. (2007). Influence of rubbing conditions of polyimide alignment layer on optical anisotropy of immobilized liquid crystal film. Liq. Cryst..

[33] Epure E.-L., Stoica I., Albu R.M., Hulubei C., Barzic A.I. (2021). New Strategy for Inducing Surface Anisotropy in Polyimide Films for Nematics Orientation in Display Applications. Nanomaterials.

[34] Stoica I., Barzic A.I., Hulubei C. (2013). The impact of rubbing fabric type on surface roughness and tribological properties of some semi-alicyclic polyimides evaluated from atomic force measurements. Appl. Surf. Sci..

[35] Luo B., Li L., Liu H., Xu M., Xing F. (2014). Analysis of Sanding Parameters, Sanding Force, Normal Force, Power Consumption, and Surface Roughness in Sanding Wood-Based Panels. BioResources.

[36] Barzic A.I., Albu R.M., Hulubei C., Thabet H.K., Abu Ali O.A., El-Bahy Z.M., Stoica I. (2022). Polyimide layers with high refractivity and surface wettability adapted for lowering optical losses in solar cells. Polymers.

[37] Barzic A.I., Stoica I., Fifere N., Vlad C.D., Hulubei C. (2013). Morphological effects on transparency and absorption edges of some semi-alicyclic polyimides. J. Polym. Res..

[38] Stoica I., Barzic A.I., Hulubei C., Timpu D. (2013). Statistical analysis on morphology development of some semialicyclic polyimides using atomic force microscopy. Microsc. Res. Tech..

[39] Kacalak W., Lipiński D., Szafraniec F., Zawada-Tomkiewicz A., Tandecka K., Królczyk G. (2020). Metrological basis for assessing the state of the active surface of abrasive tools based on parameters characterizing their machining potential. Measurement.

[40] Lemesle J., Robache F., Le Goic G., Mansouri A., Brown C.A., Bigerelle M. (2020). Surface Reflectance: An Optical Method for Multiscale Curvature Characterization of Wear on Ceramic–Metal Composites. Materials.

[41] (2012). Geometrical Product Specifications (GPS)—Surface Texture: Areal—Part 2: Terms, Definitions and Surface Texture Parameters.

[42] Lou S., Pagani L., Zeng W., Jiang X., Scott P.J. (2020). Watershed segmentation of topographical features on freeform surfaces and its application to additively manufactured surfaces. Precis. Eng..

[43] Barré F., Lopez J. (2000). Watershed lines and catchment basins: A new 3D-motif method. Int. J. Mach. Tools Manuf..

[44] Blateyron F. (2013). The Areal Field Parameters. Characterisation of Areal Surface Texture.

[45] Blateyron F. (2013). The Areal Feature Parameters. Characterisation of Areal Surface Texture.

[46] Pickering T., Shanks K., Sundaram S. (2021). Modelling technique and analysis of porous anti-reflective coatings for reducing wide angle reflectance of thin-film solar cells. J. Opt..

[47] Lee C., Kramer T.A. (2004). Prediction of three-dimensional fractal dimensions using the two-dimensional properties of fractal aggregates. Adv. Colloid Interface Sci..

[48] Boschloo G., Edvinsson T., Hagfeldt A. (2006). Dye-Sensitized Nanostructured ZnO Electrodes for Solar Cell Applications. Nanostructured Materials for Solar Energy Conversion.

[49] Mehta R., Min M., Kaul A.B. (2020). Sol-gel synthesized indium tin oxide as a transparent conducting oxide with solution-processed black phosphorus for its integration into solar-cells. J. Vac. Sci. Technol. B Nanotechnol. Microelectron. Mater. Process. Meas. Phenom..

[50] Oss C.J.V., Good R.J., Chaudhury M.K. (2002). Additive and nonadditive surface tension components and the interpretation of contact angles. Langmuir.

[51] Krč J., Zeman M., Kluth O., Smole F., Topič M. (2003). Effect of surface roughness of ZnO:Al films on light scattering in hydrogenated amorphous silicon solar cells. Thin Solid Films.

[52] Scholtz L., Ladanyi L., Mullerova J. (2015). Influence of Surface Roughness on Optical Characteristics of Multilayer Solar Cells. Adv. Electr. Electron. Eng..

[53] Brongersma M.L., Cui Y., Fan S. (2014). Light management for photovoltaics using high-index nanostructures. Nat. Mater..

[54] Kim E., Cho Y., Sohn A., Hwang H., Lee Y.U., Kim K., Park H.-H., Kim J., Wu J.W., Kim D.-W. (2016). Mie Resonance-Modulated Spatial Distributions of Photogenerated Carriers in Poly(3-hexylthiophene-2,5-diyl)/Silicon Nanopillars. Sci. Rep..

[55] Garnett E.C., Ehrler B., Polman A., Alarcon-Llado E. (2021). Photonics for Photovoltaics: Advances and Opportunities. ACS Photonics.

[56] Tonkaev P., Kivshar Y. (2020). High-Q Dielectric Mie-Resonant Nanostructures (Brief Review). JETP Lett..

